# Supplementary material for: The effect of flow on swimming bacteria controls the initial colonization of curved surfaces
Source: Nat Commun. 2020 Jun 5;11:2851. doi: 10.1038/s41467-020-16620-y (PMC7275075; doi:10.1038/s41467-020-16620-y)
Supplement: Supplementary file 1 — Supplementary Information [file 41467_2020_16620_MOESM1_ESM.docx]

**Supplementary Information**

The effect of flow on swimming bacteria controls

the initial colonization of curved surfaces

Secchi *et al.*


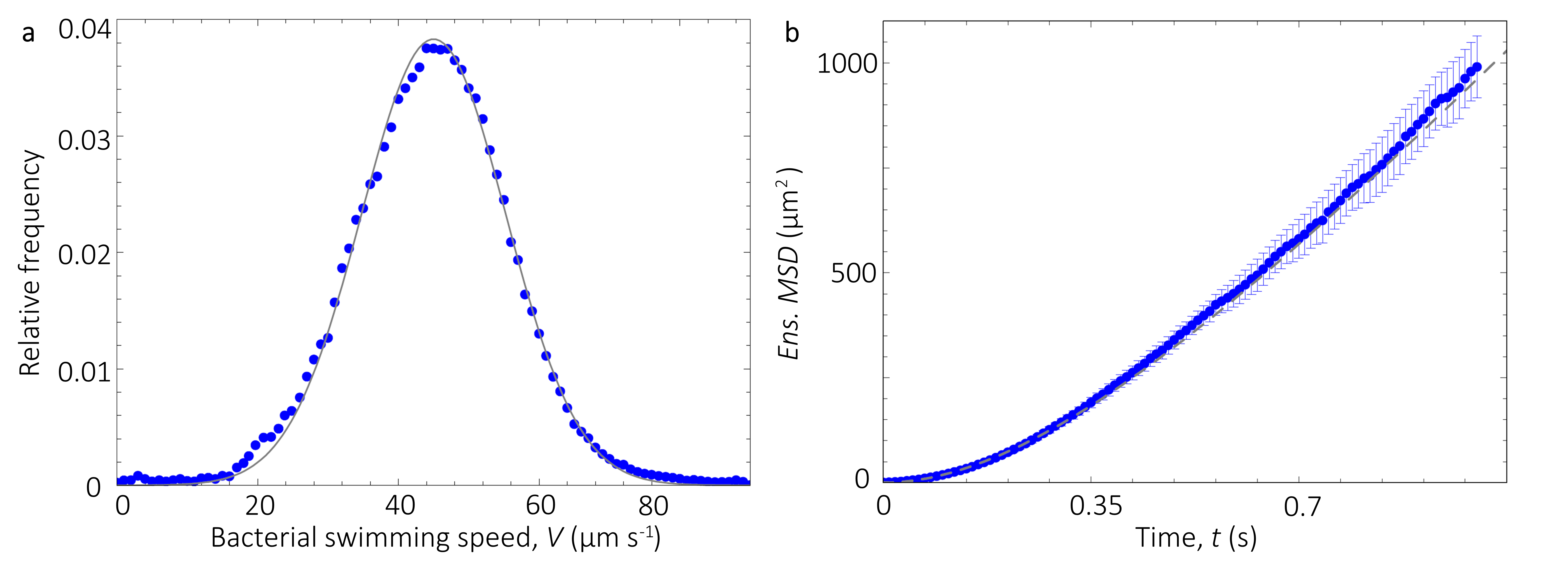


**Supplementary Figure 1**

**Measured swimming properties of *P. aeruginosa***. **a** Distribution of swimming velocities (blue circles) measured by tracking individual cells in a population of wild-type *P. aeruginosa*. The gray curve is a Gaussian fit, which yields a mean swimming speed of *V* = 45.02 µm s^-1^ with standard deviation 10.25 µm s^-1^. **b** Ensemble mean-square displacement (*MSD*) for measured cells for the same bacterial population (blue circles). The ensemble average was computed over >1500 cells tracked during three different experiments and then averaged. The gray dashed curve is a fit using the formula *MSD =* 0.5 (*V* ^2^*/D*_R_*^2^)* [2*D*_R_*t +* exp*(-*2*D*_R_*t) -* 1], which yields a rotational diffusivity of *D*_R_ = 1.4 rad^2^ s^-1^.

**
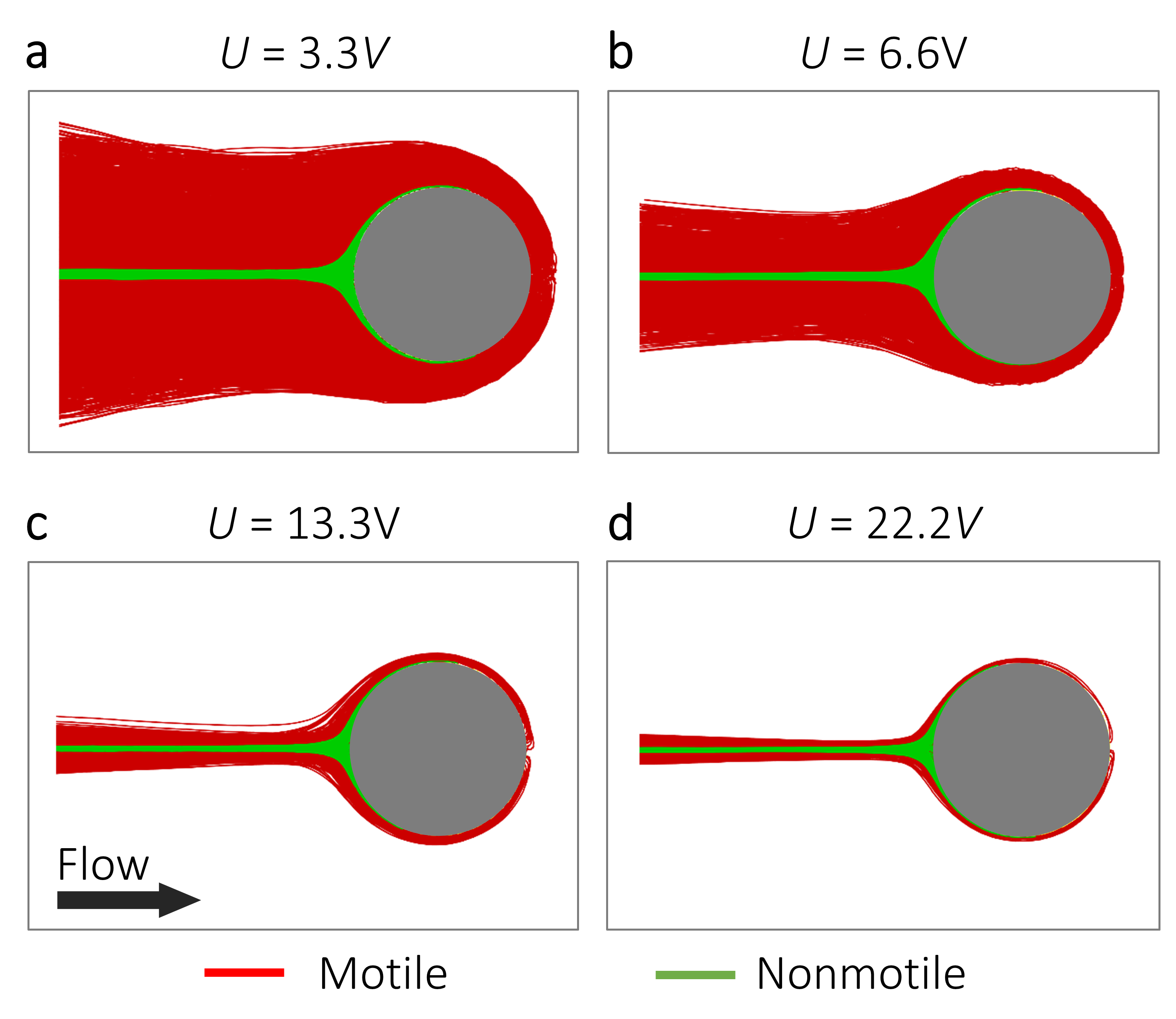
**

**Supplementary Figure 2**

**Bacterial motility dramatically increases the capture area in flow. a-d** Trajectories that encountered the 100-µm pillar, obtained with the model for motile (red) and nonmotile (green) cells for a mean flow velocity *U/V* = 3.3 (a), *U/V* = 6.6 (b), *U/V* = 13.3 (c) and *U/V* = 22.2 (d).


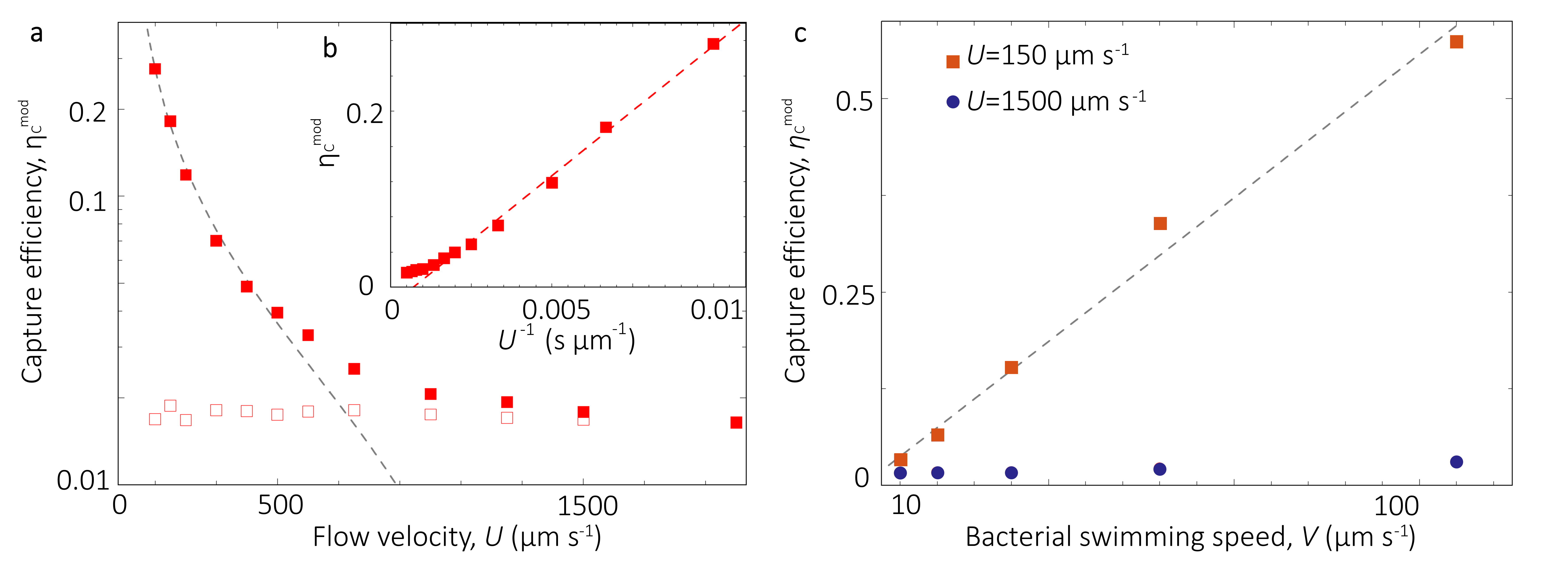


**Supplementary Figure 3**

**Capture efficiency as a function of flow velocity and bacterial swimming speed. a** Capture efficiency, ${\eta_{C}}^{\mathrm{mod}}$, as a function of the flow velocity, *U*, for a 100-µm pillar obtained from the model for motile (filled squares) and nonmotile (open squares) cells. The gray dashed curve shows the scaling ${\eta_{C}}^{\mathrm{mod}}\sim U^{-1}.$ **b** Capture efficiency, ${\eta_{C}}^{\mathrm{mod}}$, as a function of the inverse of the flow velocity, *U*^-1^. The red dashed curve shows the relationship ${\eta_{C}}^{\mathrm{mod}}\sim U^{-1}.$ **c** Capture efficiency, ${\eta_{C}}^{\mathrm{mod}}$, as a function of the bacterial swimming speed, *V*, for a 100-µm pillar obtained from the model at two values of imposed mean flow velocity, *U*. The gray dashed curve shows the scaling ${\eta_{C}}^{\mathrm{mod}}\sim V.$


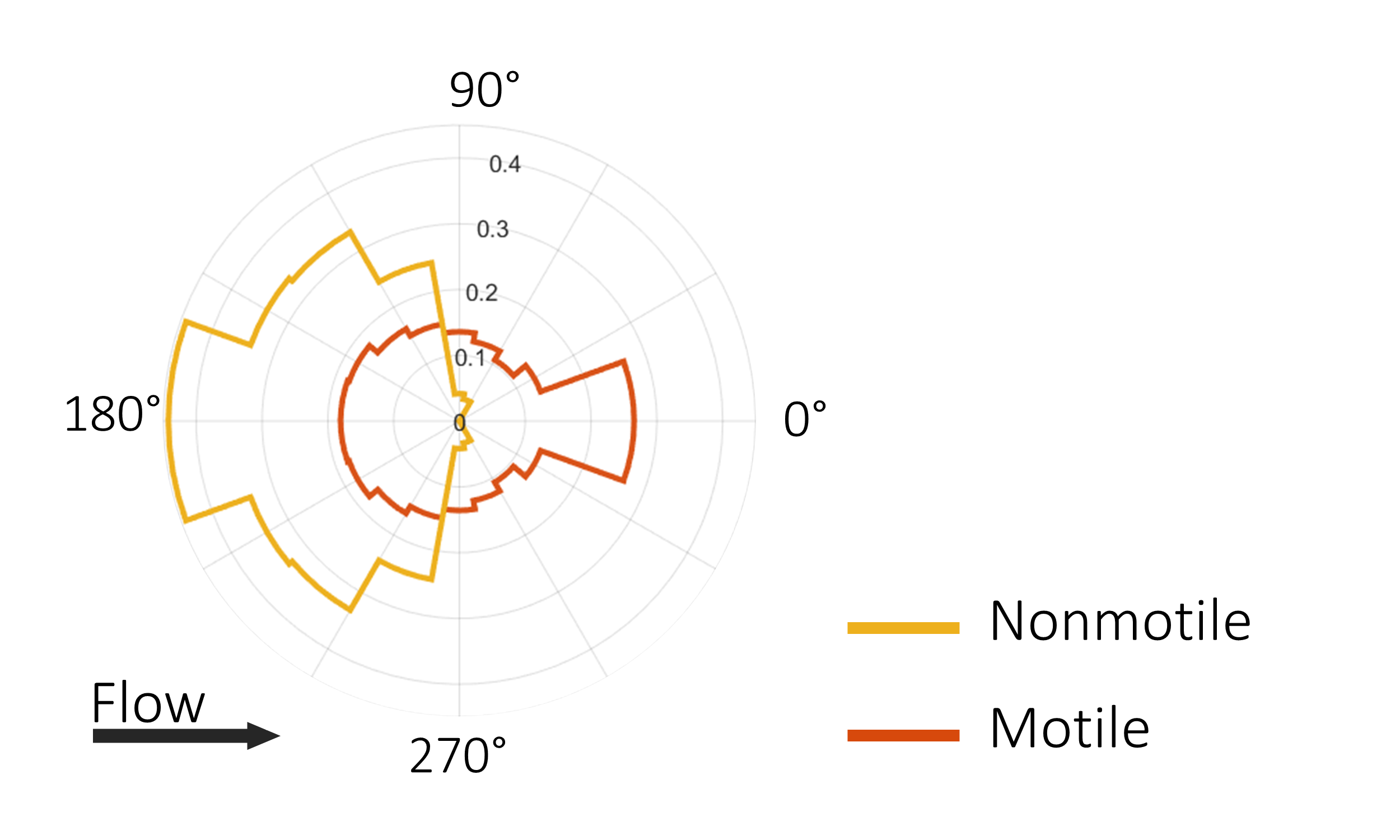


**Supplementary Figure 4**

**Bacterial motility determines the angular distribution of the attachment of bacterial the attachment of bacteria around the pillar at low flow velocity.** Angular distribution of the attachment of motile (orange) and nonmotile (yellow) elongated (*q* = 9.4) bacteria, predicted by the mathematical model for a relative flow speed of *U/V* = 6.6 and a 100-µm-diameter pillar.


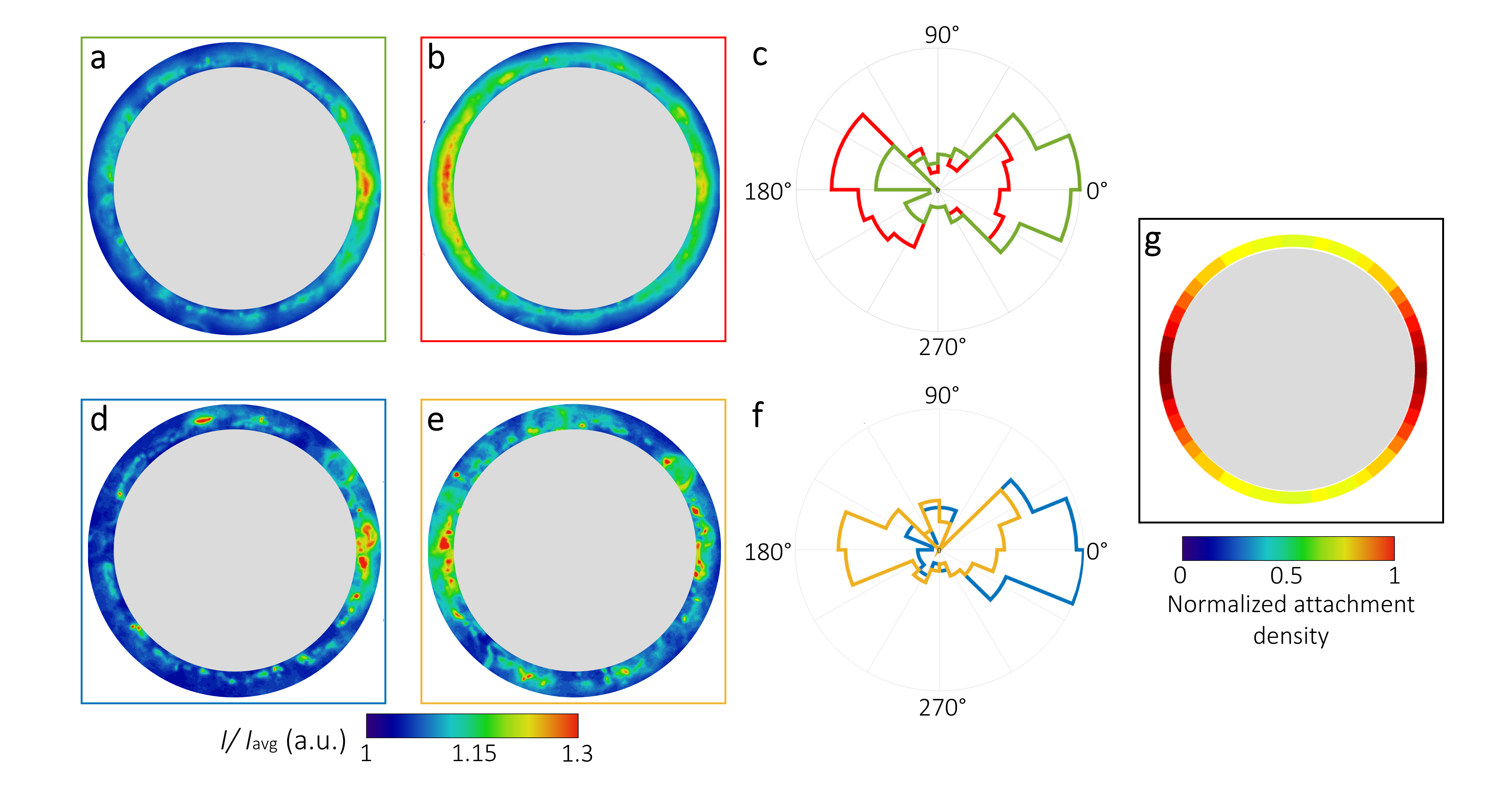


**Supplementary Figure 5**

**A flow reversal causes a reversal in the location of bacterial attachment. a** Distribution of fluorescent intensity, *I*, normalized by its mean value, *I*_avg_, after 2.5 h of flow from left to right of a diluted suspension of PA14 *wt* GFP cells in Tryptone Broth, for *U/V* = 6.6 and a 100-µm-diameter pillar. Note the leeward attachment peak on the right side of the pillar. **b** Same, but after a further 2.5 h of flow from right to left. Note the leeward attachment peak on the left of the pillar. Each intensity distribution is the average over 12 identical pillars. **c** Angular distribution of the fluorescence intensity, *I*, for the cases shown in panels a (green) and b (red). **d, e** Same as a and b, respectively, but for cells in AB minimal medium. Each intensity distribution is the average over 6 identical pillars. **f** Angular distribution of the fluorescence intensity, *I*, for the cases shown in of the panels d (blue) and e (yellow). **g** Angular distribution of the normalized attachment density of bacteria on the pillar predicted by the mathematical model in the same flow conditions as panel b, *i.e*., 2.5 h of left-to-right flow followed by 2.5 h of right-to-left flow. Simulations confirm that flow reversal causes a reversal in the location of bacterial attachment.


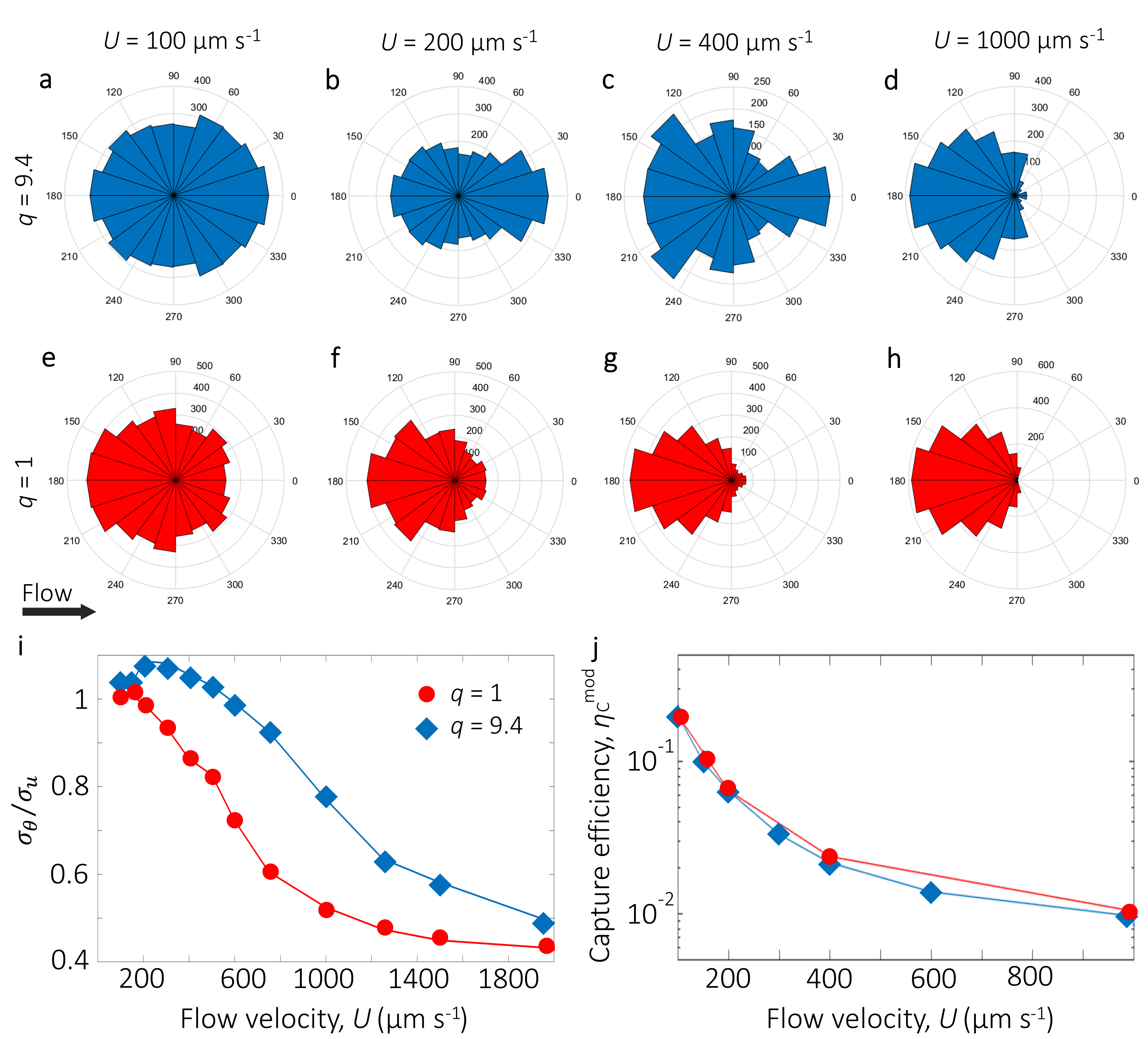


**Supplementary Figure 6**

**Attachment distribution and capture efficiency for elongated and spherical motile bacteria. a-h** Polar distribution of the attachment density of motile elongated (*q* = 9.4; a-d) and spherical (*q* = 1; e-h) cells on a 100-µm pillar at mean flow velocity of 100 µm s^-1^ (2.2*V;* a, e), 200 µm s^-1^ (4.4*V;* b, f), 400 µm s^-1^ (8.8*V;* c, g) and 1000 µm s^-1^ (22.2*V;* d, h). **i** Normalized standard deviation ${\sigma_{\theta}}/{\sigma_{u}}$ obtained with the model as a function of the mean flow velocity, *U*, for the100-µm pillar for motile elongated (*q* = 9.4; blue diamonds) and for motile spherical cells (*q* = 1; red circles). **j** Capture efficiency, *η*_C_^mod^, as a function of the mean flow velocity, *U*, for a 100-µm pillar obtained from the model for motile elongated (*q* = 9.4; blue diamonds) and for motile spherical cells (*q* = 1; red circles).


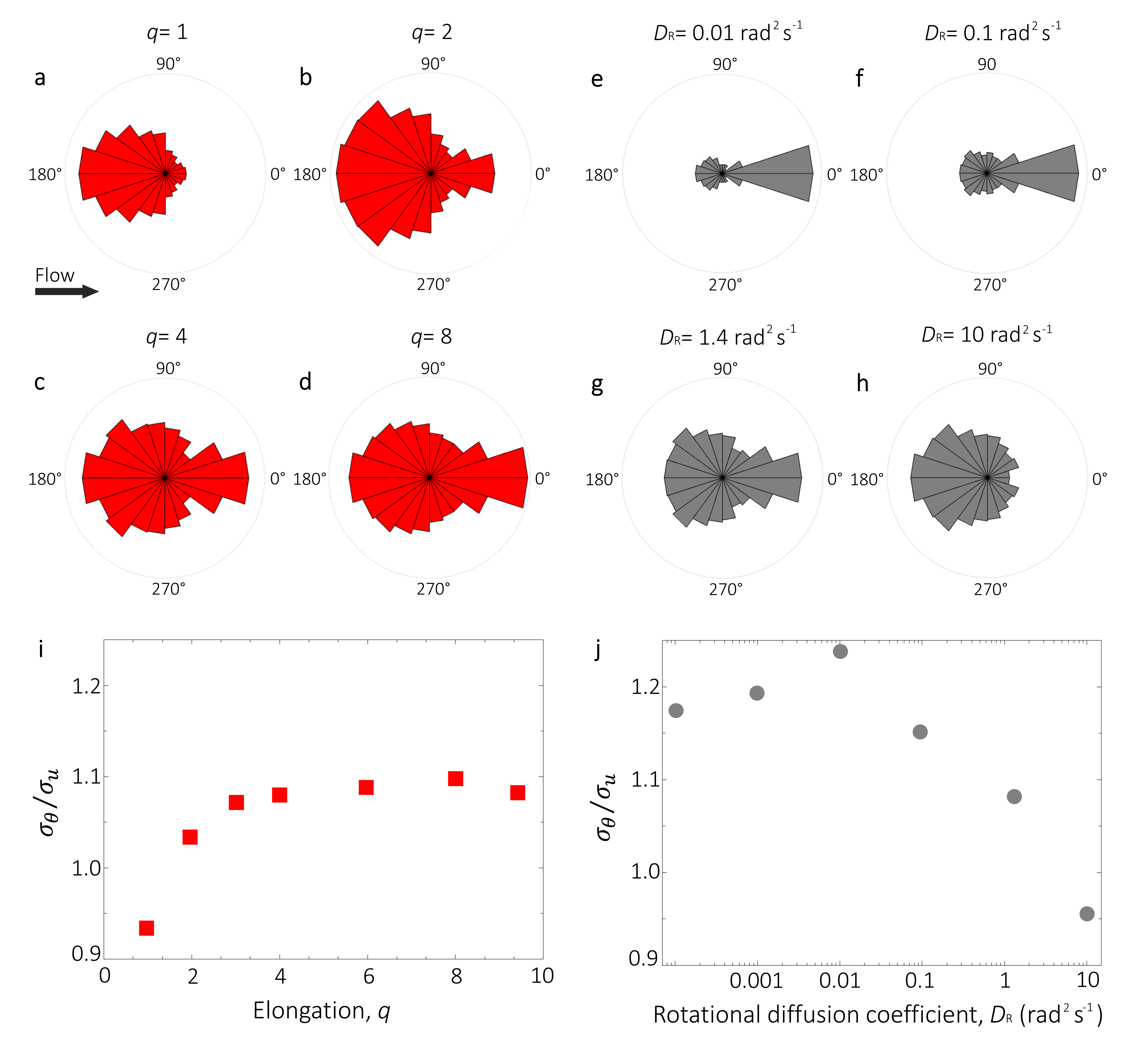


**Supplementary Figure 7**

**Angular distribution of the attachment of motile bacteria with different elongations and rotational diffusivities,** predicted by the mathematical model for a relative flow speed of *U/V* = 6.6 and a 100-µm-diameter pillar. **a-d** Polar distribution of the attachment density of cells with elongation *q* = 1 (a), 2 (b), 4 (c) and 8 (d). **e-h** Polar distribution of the attachment density of cells with rotational diffusivities *D*_R_ = 0.01 rad^2^ s^-1^ (e), 0.1 rad^2^ s^-1^ (f), 1.4 rad^2^ s^-1^ (g) and 10 rad^2^ s^-1^ (h). **i, j** Normalized standard deviation of the polar distribution, ${\sigma_{\theta}}/{\sigma_{u}}$, as a function of (i) the cell elongation *q* and (j) the cell rotational diffusivity *D*_R_.


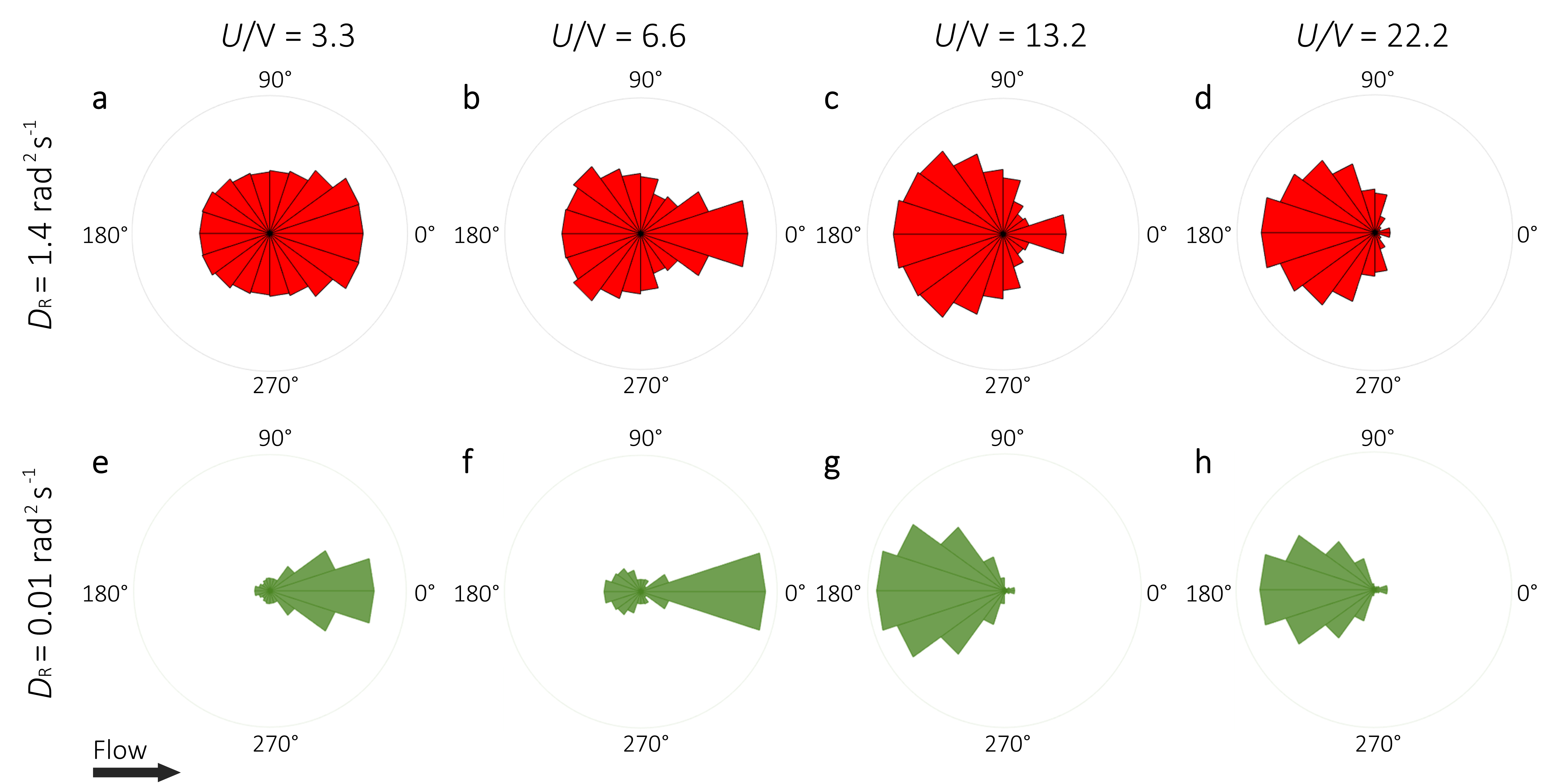


**Supplementary Figure 8**

**Angular distribution of the attachment of motile bacteria for different flow speeds *U*/*V*,** and two values of the rotational diffusivity *D*_R_ predicted by the mathematical model for a 100-µm-diameter pillar and for cells with elongation *q* = 9.4.


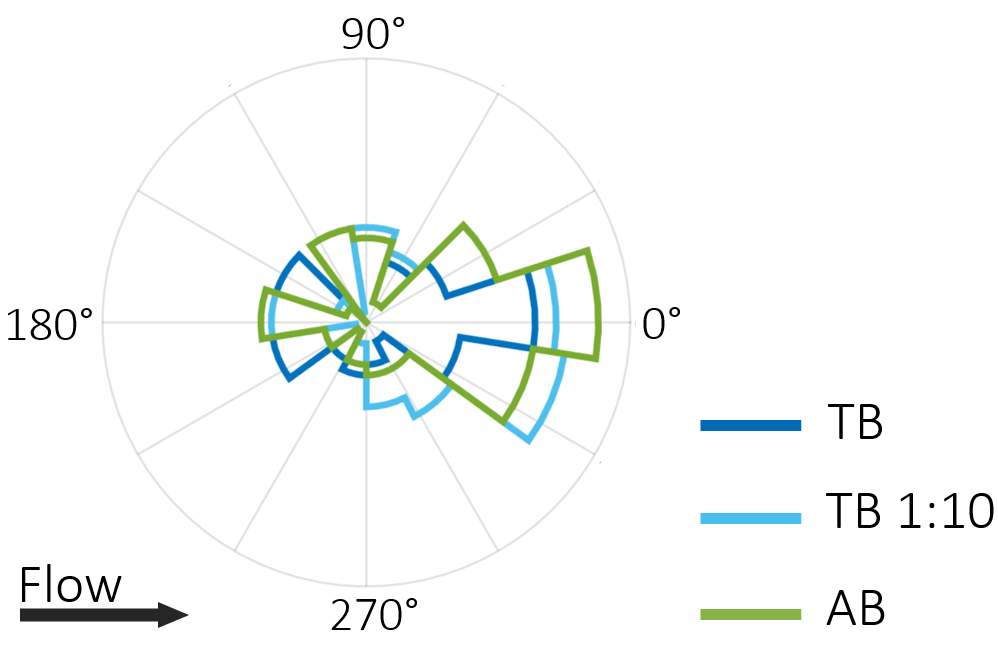


**Supplementary Figure 9**

**The culture medium does not affect the angular distribution of bacterial colonization around a pillar.** Angular distribution of the fluorescence intensity, *I*, on a 100-µm pillar after 5 h of flow at *U/V* = 6.6 of a diluted suspension of PA14 *wt* GFP cells in different culture media: Tryptone Broth (TB; blue), Tryptone Broth diluted 1:10 in an isotonic saline solution (TB 1:10, cyan) and AB medium (AB, green). Each intensity distribution is the average over 12 identical pillars.


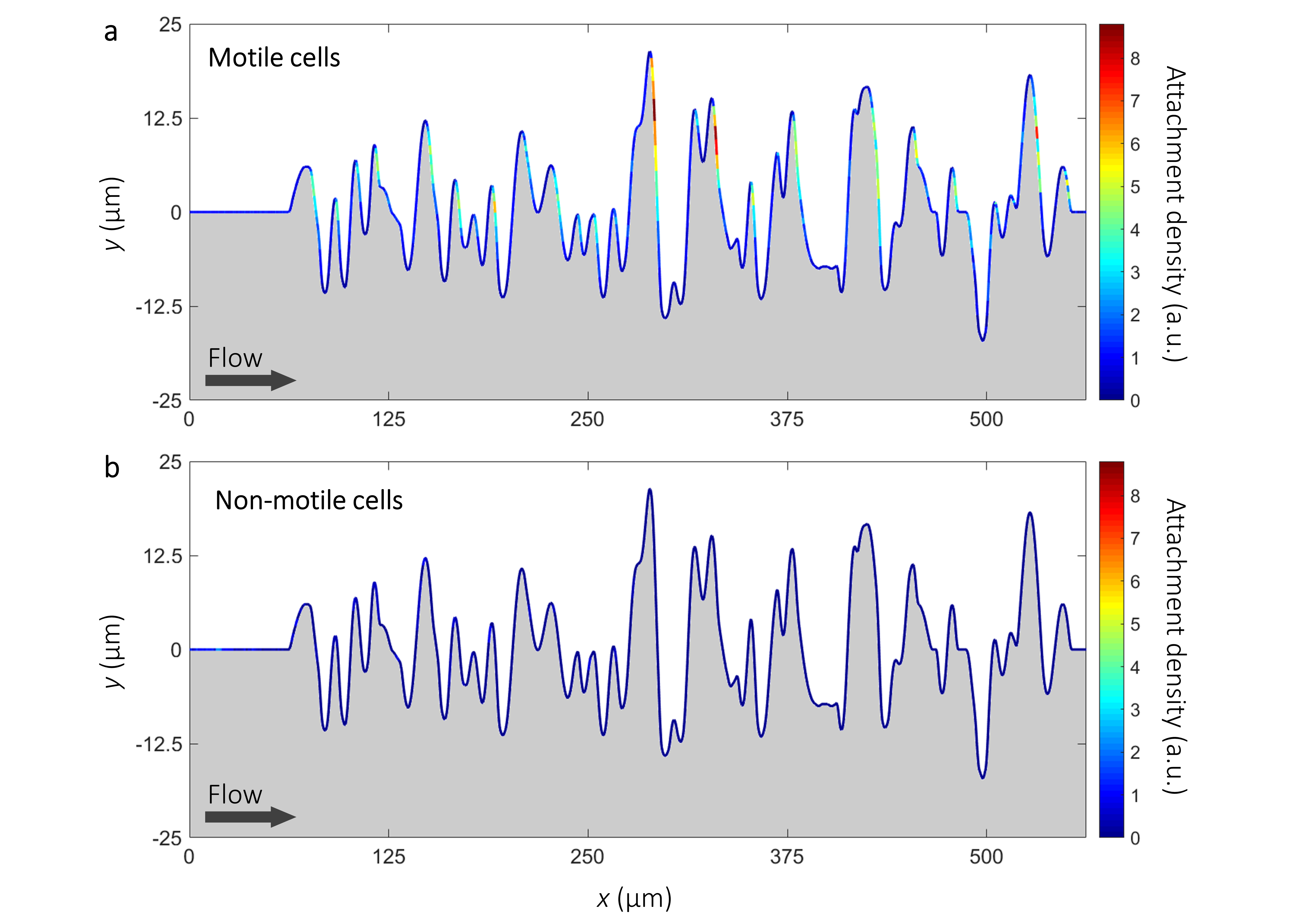


**Supplementary Figure 10**

**Colonization of a random corrugated surface by motile and nonmotile cells.** Normalized attachment density of motile (*q* = 8.5, *V* = 21.6 μm s^-1^; a) and nonmotile (*q* = 8.5, *V* = 0 μm s^-1^; b) cells on a random corrugated surface obtained with the model at a mean flow velocity of 150 µm s^-1^.

**Supplementary Methods**

**Culture preparation and media**. The following procedure was used immediately prior to experiments in order to obtain motile cells, according to [1]. 1.5 mL of culture medium was added to a single well of a 6-well cell culture plate (BD Biosciences, San Jose, CA). A sterile cell culture insert (BD Biosciences, San Jose, CA), incorporating a polyethylene terephthalate (PET) track-etched membrane (pore size: 3 μm), was placed into the well and wetted with the culture medium, while avoiding trapped air under the membrane. 1 mL of cultured bacterial solution was then added to the insert. The bacterial culture was allowed to stand for 15 min, during which time motile bacteria migrated through the membrane, while nonmotile cells settled onto the membrane surface. The pore size of the membrane was chosen to allow the passage of only single cells. The filter insert was gently removed and the remaining solution in the well, containing a high fraction of motile cells, was diluted to OD_600_ = 0.01 and used in experiments.

AB medium was prepared according to the following protocol [2]: 200 mL of a 5xA solution (add 2 g of (NH_4_)_2_SO_4_, 6 g of Na_2_HOP_4_, 3 g of KH_2_PO_4_ and 3 g of NaCl to 200 mL of deionized water; mix and autoclave) and 800 mL of 1xB solution (add 1 mL of a 0.1 M CaCl_2_ sterile solution, 1 mL of a 1 M MgCl_2_ sterile solution and 1 mL of a 0.003 M FeCl_3_ sterile solution to 797 mL of sterilize deionized water).

**Bacterial GFP tagging**. A green fluorescent protein (GFP) [3] was PCR amplified using pGFP_Fw_XbaI (tcctctagaGCGGCCGCTCTAGACATT) and pGFP_Rv_KpnI (ttcggtaccGTACCCAGCTGTTGACTCG). The amplicon was cleaned (QIAquick PCR Purification kit, Cat No./ID:28106), cut with KpnI_HF (New England Biolabs, Cat No. R3142S) and XbaI (New England Biolabs, Cat No. R0145S) and ligated into the plasmid pBBR1MCS-3 [4] digested with the same enzymes. To mark the *P. aeruginosa* PA14 wild-type and mutant strains (*motB* and *flgE*) [5] with GFP, plasmid pBBR1MCS-3::gfp was mobilized into each strain by triparental mating as described previously [6] using the helper *E. coli* pRK2013 [7].

**Microfluidic assay**. Microfluidic channels were fabricated using standard soft lithography techniques. Microchannel molds were prepared by depositing SU-8 2150 (MicroChem Corp., Newton, MA) on silicon wafers via photolithography. Polydimethylsiloxane (PDMS; Sylgard 184 Silicone Elastomer Kit, Dow Corning, Midland, MI) was prepared (with 10% by weight of cross-linker) and cast on the molds. After curing, PDMS microchannels were plasma-sealed onto a clean glass slide. Channels were flushed with 2 ml of fresh medium before each experiment.

**Particles’ diffusion coefficient**. The particles’ diffusion coefficient was computed as follows. For Brownian particles, the diffusion coefficient is given by the well-known Stokes-Einstein relation, i.e., *D = D*_0_ *= k*_b_*T ⁄* 6*πηR*, where *k*_b_ is the Boltzmann constant, *T* is the temperature of the system, *η* is the fluid viscosity and *R* is the particle radius. The random walk-like swimming behavior of motile bacteria allows the definition of an effective diffusion coefficient that is expressed as *D = D_0_ + (V*^2^ *τ*_R_*) ⁄* 4, where *V* is the bacterial swimming speed and *τ*_R_ is the rotational diffusional time [8].

**Langevin model of bacterial motility in flow**. We assumed that the fluid forces on bacteria were governed by the Stokes flow conditions. The effective aspect ratio of a *P. aeruginosa* cell, composed of a 2.4 μm by 1.2 μm ellipsoidal bacterial cell body and 10-μm-long helical flagellar bundle, used in the model was *q* = 9.4; the effective aspect ratio of a *E. coli* cell, composed of a 1 μm by 0.5 μm ellipsoidal bacterial cell body and 8-μm-long helical flagellar bundle, used in the model was *q* = 8.5. These values were computed from resistive force theory. Briefly, we computed the hydrodynamic resistance coefficients of the composite structure (cell and flagellar bundle) and then determined the aspect ratio, *q*, of an ellipsoid having the same rotational mobility in shear (i.e., the same Jeffery orbit). The cell swimming speed used in the model, *V* = 45 μm s^-1^ for *P. aeruginosa* (Supplementary Fig. 1) and *V* = 21.6 μm s^-1^ for *E. coli*, and rotational diffusivity, *D*_R_ = 1.4 rad^2^ s^-1^ for *P. aeruginosa* and *D*_R_ = 0.32 rad^2^ s^-1^ for *E. coli*, were directly measured by tracking individual cells in the absence of flow (for more details see [1]).

**Supplementary References**

[1] Rusconi R, Guasto JS, Stocker R. Bacterial transport suppressed by fluid shear. *Nat Phys* **10**, 212–217 (2014).

[2] Jensen KF. The Escherichia coli K-12 "wild types" W3110 and MG1655 have an rph frameshift mutation that leads to pyrimidine starvation due to low pyrE expression levels. *J Bacteriol.* **175**, 3401-3407 (1993).

[3] Lambertsen, L., Sternberg, C. & Molin, S. Mini-Tn7 transposons for site-specific tagging of bacteria with fluorescent proteins. *Environ. Microbiol*. **6**, 726–732 (2004).

[4] Kovach, M. E. et al. Four new derivatives of the broad host range cloning vector PBBR1MCS, carrying different antibiotic resistance cassettes. *Gene* **166**, 175–176 (1995).

[5] Liberati, N. T. et al. An ordered, nonredundant library of *Pseudomonas aeruginosa* strain PA14 transposon insertion mutants. *Proc. Natl. Acad. Sci. USA* **103**, 2833–8 (2006).

[6] Huber, B. et al. Genetic analysis of functions involved in the late stages of biofilm development in *Burkholderia cepacia* H111. *Mol. Microbiol*. **46**, 411–426 (2002).

[7] Figurski, D. H. & Helinski, D. R. Replication of an origin-containing derivative of plasmid RK2 dependent on a plasmid function provided in trans. *Proc. Natl. Acad. Sci*. *USA* **76**, 1648–1652 (1979).

[8] Howse JR, et al. Self-motile colloidal particles: from directed propulsion to random walk. *Phys Rev Lett* **99**, 8–11 (2007).
